# Compressed sensing with synchronized cardio-respiratory sparsity for free-breathing cine MRI: initial comparative study on patients with arrhythmias

**DOI:** 10.1186/1532-429X-16-S1-O17

**Published:** 2014-01-16

**Authors:** Li Feng, Leon Axel, Larry A Latson, Jian Xu, Daniel K Sodickson, Ricardo Otazo

**Affiliations:** 1 Bernard and Irene Schwartz Center for Biomedical Imaging, New York University School of Medicine, New York, New York, USA; 2 Siemens Medical Solutions, New York, New York, USA

## Background

Evaluation of myocardial function with MRI is challenging on patients with impaired breath-hold (BH) capabilities or arrhythmias due to the difficulty of respiratory motion suspension and synchronization of cardiac cycles. Compressed sensing (CS) enables free breathing (FB) real-time cine imaging with improved spatiotemporal resolution, but conventional temporal sparsifying transforms do not account for respiratory motion, which limits its performance. In this work, we propose to acquire data continuously in FB using a golden-angle radial sampling scheme and reconstruct images with separated but synchronized cardiac and respiratory motion dimensions using self-detected motion signals. For patients with arrhythmias, both "normal" and "ectopic" cycles are reconstructed by sorting out cardiac cycles with different lengths. The performance of the proposed method was compared to Cartesian BH approach using retrospective ECG-gating in 9 patients.

## Methods

**Figure 1:**
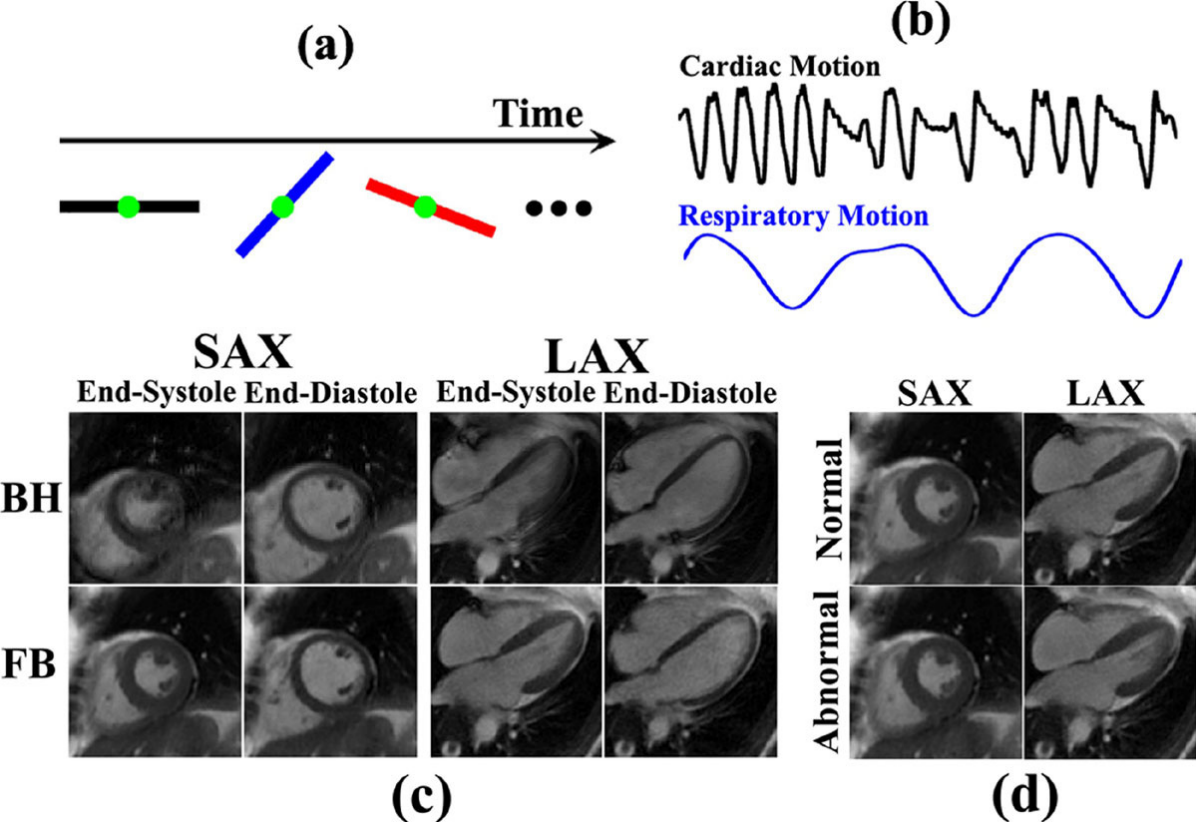
**(a) Continuous data acquisition**. (b) Cardiac/respiratory motion detection in the presence of arrhythmias. (c) Comparison between breath-hold cine imaging with retrospective ECG-gating and free-breathing cine imaging with cardio-respiratory synchronization in a patient with Mobitz I. (d) Reconstruction of free-breathing end-systolic cardiac phases from both normal and abnormal cardiac cycles.

Both BH and FB cine sequences (b-SSFP) were implemented on a 1.5T MRI scanner (Avanto, Siemens). Imaging parameters for BH cine were: spatial resolution = 1.8 × 1.8 mm^2^, slice thickness = 8 mm, TR/TE = 2.5/1.25 ms, FA = 55°. Imaging parameters for FB cine were: spatial resolution = 2 × 2 mm^2^, slice thickness = 8 mm, TR/TE≈2.8/1.4 ms, FA = 70°. Both sequences achieved temporal resolution ~30-40 ms. Cardiac imaging was performed on 9 patients (mean age = 56; 4 had normal sinus rhythm, 4 had arrhythmias including bigeminy PVCs, atrial fibrillation and Mobitz I, 1 was incapable of prolonged BH). One short axis and one 4 chamber cine image set were acquired on each patient at ~12-15s per slice. In FB cine imaging, central k-space positions (green dots, Figure 1a) were used to extract cardiac and respiratory signals from coils near the heart and diaphragm respectively (Figure 1b). Data were sorted and synchronized to separately reconstruct cardiac cycles of different lengths at different respiratory states. A multicoil CS approach was used to reconstruct the undersampled datasets, using a total variation constraint along both cardiac and respiratory dimensions. Images were randomized and blinded for radiologist evaluation (1-5; worst-best). Statistical analysis was performed to compare mean scores of BH and FB within two groups, one including normal sinus rhythm and the other including arrhythmias or impaired BH capability.

## Results

Figure 1c shows BH and FB results on one patient with Mobitz I. The proposed approach produced better image quality due to the ability to differentiate "normal" and "ectopic" cycles (Table 1, group 2). For patients with normal sinus rhythm, both approaches produced good image quality (Table 1, group 1). Figure 1d shows FB cine reconstructions from "normal" and "ectopic" cycles.

**Table 1:** Image quality comparison between breath-hold and free-breathing cine images.

Technique	Group 1	Group 2
BH	3.75 ± 0.46	2.0 ± 0.93
FB	3.0 ± 0.53	3.1 ± 0.74

Group 1 included patients with normal sinus rhythm and group 2 included patients with arrhythmias or impaired breath-hold capability.

## Conclusions

Separation of cardiac and respiratory motion into different dimensions improves compressed sensing reconstruction for free-breathing imaging. Additional physiological information can be obtained by separately reconstructing cardiac cycles of different lengths.

## Funding

National Institutes of Health: R01 EB000447.

